# Diet supplementation with the macroalgae *Laurencia glandulifera* and *Dictyopteris membranacea* limits postprandial hyperglycemia and alters intestinal microbiome in mice

**DOI:** 10.3389/fnut.2026.1923067

**Published:** 2026-08-04

**Authors:** Maria G. Daskalaki, Ahmed A. Al-Qahtani, Antiopi Tsoureki, Evangelia Mouchtaropoulou, Avery E. Hurst, Stefanos Kikionis, Maria Harizani, Fatimah Alhamlan, Hani Alothaid, Antonios M. Makris, Sotirios C. Kampranis, Anagnostis Argiriou, Vassilios Roussis, Efstathia Ioannou, Christos Tsatsanis

**Affiliations:** 1 Laboratory of Clinical Chemistry, Medical School, University of Crete, Heraklion, Greece; 2 Institute of Molecular Biology and Biotechnology, FORTH, Heraklion, Greece; 3 Department of Infection and Immunity, Research Center, King Faisal Specialist Hospital and Research Center, Riyadh, Saudi Arabia; 4 Department of Microbiology and Immunology, College of Medicine, Alfaisal University, Riyadh, Saudi Arabia; 5 Institute of Applied Biosciences, Center for Research and Technology Hellas, Thermi, Thessaloniki, Greece; 6 Department of Food Science and Nutrition, University of the Aegean, Myrina, Lemnos, Greece; 7 Section of Pharmacognosy and Chemistry of Natural Products, Department of Pharmacy, National and Kapodistrian University of Athens, Panepistimiopolis Zografou, Athens, Greece; 8 Department of Basic Medical Sciences, Faculty of Applied Medical Sciences, Al-Baha University, Al-Baha, Saudi Arabia; 9 Section of Plant Biochemistry, Department of Plant and Environmental Sciences, University of Copenhagen, Frederiksberg, Denmark

**Keywords:** dictyopteris, dietary supplements, inflammation, intestinal microbiome, laurencia, macroalgae, obesity

## Abstract

**Background:**

Development of obesity and type 2 diabetes largely depends on dietary habits that concomitantly affect the gut microbiome resulting in dysbiosis. Several dietary supplements have been suggested to suppress development of diabetes, partly via changes in the gut microbiome. Earlier research has shown that secondary metabolites from the marine algae *Laurencia glandulifera* and *Dictyopteris membranacea*, possess anti-inflammatory properties suppressing intestinal inflammation *in vivo*. Since these algae are part of the local community diet, we aimed to investigate their potential use as dietary supplements with beneficial effect in preventing early obesity and development of glucose intolerance. Their impact in modulating the gut microbiome was also evaluated, being readily affected by diet.

**Methods:**

We utilized the mouse model of diet-induced type 2 hyperglycemia applying a 4-week early obesity protocol, supplementing animal feed with dried mass of the marine algae *Laurencia glandulifera* and *Dictyopteris membranacea*. Intestinal microbiome changes were assessed using 16S sequencing, inflammatory profile was examined by measuring cytokine, chemokine and adipokine in serum and adipose tissue, whereas glucose intolerance was verified by glucose tolerance test.

**Results:**

Dietary supplementation with either of the two algae limited postprandial hyperglycemic spikes and induced colonization of distinct microbiota, partially reversing some of the changes induced by high fat diet, including reducing colonization of *Helicobacter* and promoting colonization of beneficial species *Dubosiella* and *Akkermansia*. In addition, both algae significantly reduced expression of the proinflammatory cytokines IL-6, IL-12 and chemokines cxcl1 and cxcl2 in the adipose tissue. *L. glandulifera* reduced weight gain of mice during the early stages of the model, whereas *D. membranacea* did not, although mice from both groups exhibited reduced leptin expression.

**Conclusion:**

These findings suggest that dietary supplementation with *L. glandulifera* or *D. membranacea* promotes beneficial changes in the gut microbiome and suppresses metabolic inflammation and postprandial hyperglycemia at the early stages of obesity, paving the way to further elucidate the underlying mechanism.

## 1 Introduction

Obesity has been steadily increasing in the past decades, reaching epidemic proportions in developed countries. According to the latest World Health Organization data, over 1 billion adults worldwide are living with obesity whereas childhood obesity is also accelerating globally, with over 1 in 5 children being overweight or obese. These alarming statistics reflect the potential implications of obesity in public health, since obesity is associated with numerous pathological conditions, including metabolic syndrome, cardiovascular diseases, type 2 diabetes, and cancer (1, 2).

Obesity is a chronic, complex disease characterized by excessive fat deposits, giving rise to obesity-related complications, such as the induction of type 2 diabetes and metabolic syndrome (3). In obesity and type 2 diabetes, low-grade systemic inflammation is induced, impairing insulin signaling and/or secretion, resulting in insulin resistance and hyperglycemia, and causing metabolic rewiring and inflammatory cell dysregulation (4). Although it can be linked to genetic mutations and epigenetic changes, obesity is a multifaceted condition influenced by modern lifestyle, dietary habits, lack of physical exercise, and microbiome dysregulation (2). According to recent evidence, alterations in the composition and function of the intestinal microbiome, known as dysbiosis, can both disrupt the host's gut barrier, as well as dysregulate immune and metabolic functions via inflammasomes, Toll-like receptor (TLR) signaling, and glucose and lipid metabolism (5). Dietary interventions using specialized diets and supplementation with bioactive compounds show direct benefits in combating obesity and type-2 diabetes (6). In obesity research, animal model studies are of immense importance in understanding the impact of dietary interventions in disease establishment and progression. In particular, high-fat diet (HFD)-induced obesity in mice is a well characterized and accepted mouse model used for studying obesity and type 2 diabetes.

Natural products represent the richest resource of bioactive compounds, exhibiting a very wide spectrum of biological properties, such as anticancer, antibacterial, antifungal, anti-inflammatory, and antioxidant, among others. In recent years, marine organisms have attracted significant interest as sources of health-promoting com-pounds. Among those, marine macroalgae distributed worldwide have been extensively studied for their chemical constituents resulting in the characterization of more than 5,000 secondary metabolites, many of which have shown significant levels of bioactivity (7). Because of their high nutritional value and abundance of bioactive compounds, macroalgae and microalgae have both become promising functional food ingredients. While macroalgae are especially rich in structurally diverse polysaccharides, polyphenols, and unique secondary metabolites with anti-inflammatory and prebiotic properties, microalgae have garnered significant attention due to their protein, carotenoid, and polyunsaturated fatty acid content and ease of cultivation compared to macroalgae. Furthermore, many coastal populations have long directly consumed edible macroalgae, which makes them appealing options for dietary supplements meant to enhance metabolic health and alter the gut microbiota (8, 9). In the East Mediterranean, red algae of the genus Laurencia and the brown alga *Dictyopteris membranacea* are often found in dense populations and are consumed as a delicacy by local coastal communities. In previous studies, we have isolated, among others, brominated diterpenes (1–3) from *Laurencia* sp., collected in the island of Kefalonia, and disulfides (4–6) from *D. membranacea*, collected from various locations along the Greek coastline that have been shown to possess anti-inflammatory and analgesic properties in mice, reducing TLR4-induced inflammation and DSS-induced intestinal inflammation (7, 10, 11).

**Figure 1:**
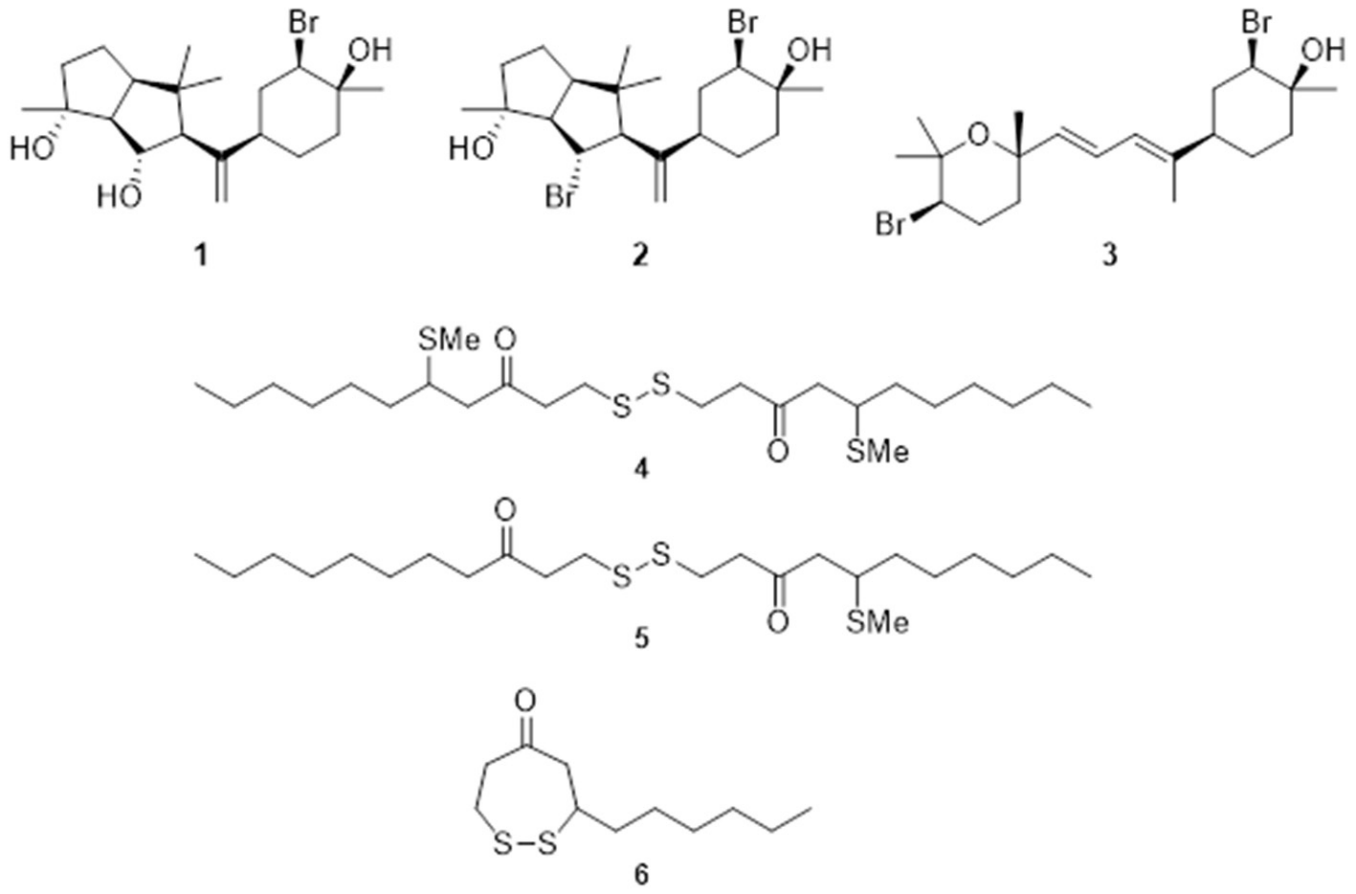
Chemical structures of compounds possessing anti-inflammatory activity from Laurencia sp. collected in the island of Kefalonia **(1–3)** and D. membranacea collected from various locations in Greece **(4–6)**. Adapted from Figure 1 in Daskalaki et al. (7) and Daskalaki et al. (10) under the terms and conditions of the Creative Commons Attribution (CC BY) license (http://creativecommons.org/licenses/by/4.0).

Although great interest has been referred to the discovery of new bioactive metabolites, limited evidence exists regarding the effects of whole algal biomass on early-stage metabolic dysfunction and gut microbiota modulation *in vivo*. In search of natural dietary supplements that could be used to reduce glucose intolerance, metabolic dysregulation and intestinal microbiome composition, the present study aimed to investigate the effects of dietary supplementation with dried biomass of *Laurencia glandulifera* and *Dictyopteris membranacea* in a high-fat diet-induced murine model of early-stage obesity. For this purpose, the effect of total dried algal biomass of *L. glandulifera and D. membranacea containing* 1–3 and 4–6, respectively (Figure 1), was assessed. These algae are traditionally consumed by local communities and it was hypothesized that supplementation with these macroalgae would prevent development of metabolic inflammation and glucose intolerance, and affect gut microbiota composition during high-fat diet driven obesity at its early stages of development.

## 2 Materials and methods

### 2.1 Collection of algal material

Specimens of *L. glandulifera* were collected by hand at Vatsa Bay in Kefalonia is-land, Greece (GPS coordinates 38°09′40^′′^N, 20°22′35^′′^E), at a depth of 0.5 to 2 m, in July 2018. Specimens *of D. membranacea* were collected by hand at Filiatro Bay, in Ithaki Island, Greece (GPS coordinates 38°22′18^′′^N, 20°44′40^′′^E), at a depth of 0.5 to 2 m, in August 2018. Voucher specimens of the algae were deposited at the Herbarium of the Section of Pharmacognosy and Chemistry of Natural Products, Department of Pharmacy, National and Kapodistrian University of Athens (ATPH/MP0744 and ATPH/MP0749, respectively). The algal material was thoroughly cleaned from epiphytes, washed with fresh water twice, dried in a vacuum oven at 40 °C until complete removal of moisture, and finally powdered in a Waring blender.

### 2.2 Chemical analysis of the investigated algae

Specimens of the dried algae (10 g) were exhaustively extracted with CH_2_Cl_2_/MeOH 1:1 (v/v) (100 ml for 24 h, twice) to afford the organic extracts. Following removal of the solvents under vacuum, the green-brown oily residues were analyzed by 1H NMR spectroscopy using CDCl3 as solvent and thin layer chromatography (TLC) using appropriate solvent systems and the isolated compounds 1–6 as standards. 1H NMR spectra were recorded on a Bruker DRX 400 MHz spectrometer.

### 2.3 Animal maintenance and related protocols

Animals were housed, handled and treated according to national and EU legislation on laboratory animal handling and the related protocols were approved by the University of Crete Ethics Committee (License Number 269884) (Heraklion, Crete, Greece). All authors and procedures are compliant with the ARRIVE guidelines. C57BL/6 mice were obtained from the Jackson Laboratory (USA) and were maintained in a 12h day/night cycle at 21–23 °C, in the animal facility at the Medical School of the University of Crete, Heraklion. 8-weeks old mice were used, 3 male and 3 female mice (total 6 mice) per group were used for a period of 4 weeks. Mice were group-housed in conventional cages at a density of 3 adult males or females per cage, fully compliant with institutional guidelines and the Guide for the Care and Use of Laboratory Animals with two cages per experimental group (*n* = 6 mice/group, one cage with 3 males and one cage with 3 females per group). The rationale for using both male and female mice was to identify gender/sex-independent effects in metabolic inflammation of algae supplementation, since dietary supplements target the entire population and not one gender. Four groups were included, one receiving chow diet (normal diet), one receiving HFD, one receiving HFD supplemented with dried Laurencia, and one receiving HFD supplemented with dried Dictyopteris (Table 1). Algae consuming groups were supplemented with 2% w/w algae as this amount was well tolerated by the mice and also corresponds to established protocols using marine algae supplementation in high-fat diet mouse models for metabolic and microbiome research, where 1%−5% w/w dry algae in feed is common to achieve bioactive effects without toxicity (12–15), equivalent to human supplemental intakes (16). Before initiation of the main animal experiment, a preliminary cage-based feeding study was conducted to verify that incorporation of 2% (w/w) algal biomass did not affect diet palatability or food acceptance (17).

**Table 1:** Groups of mice that were used and type of diet supplementation.

Groups	Number of mice	Diet	Supplementation
Control	6	Chow diet	None
Control + HFD	6	High-fat diet	None
Laurencia +HFD	6	High-fat diet	*L. glandulifera*
Dictyopteris + HFD	6	High-fat diet	*D. membranacea*

For the chow diet group, mice had free access to standard maintenance feed, Mucedola 4RF21 3% fat, whereas for HFD groups mice had free access to special diet feed, Mucedola PF4215, 60% energy from fat. Their composition is shown in Table 2. Glucose tolerance test was conducted at 3.5 weeks following initiation of the experiment. Mice were fasted overnight and in the morning glucose measurements were conducted. The basal glucose levels were referred to as timepoint 0. Mice were then injected intraperitoneally with sterile 35% dextrose (100 μL dextrose/35 g mice) and glucose levels were determined 30-, 60-, and 120-min post injection in whole blood specimen. Glucose was measured with the TRUEresult^®^ Twist meter (Nipro diagnostics, Fort Lauderdale, FL, USA). At the end of the experimental protocol, mice were euthanized through cervical dislocation.

**Table 2:** Diet composition.

Component	Chow (4RF21)	HFD (PF4215)
Energy (kcal/kg)	~3,876	5,500–6,000
Fat (%)	3.0	34
Protein (%)	18.5	23
Carbohydrates (%)	53.5	38
Fiber (%)	6.0	5
Ash (%)	7.0	5.5

Chow diet purchased from Mucedola Cat No. 4RF21, consists of (in descending order of inclusion): Wheat, Maize, Soybean meal extracted toasted, Corn, gluten feed, Wheat straw, Fish meal, Lucerne meal, Mineral dicalcium phosphate, Calcium carbonate, Sodium chloride, Whey powder, Soybean oil, Yeasts.

### 2.4 RNA extraction, cDNA synthesis, and real-time PCR

Following animal sacrifice, visceral white fat tissue was collected, weighed, and stored at −80 C before nucleic acid extraction. Specifically, sterile forceps were used to isolate perigonadal adipose tissue from mice mice's, periovarian in females and epididymal in males. Adipose tissue was homogenized in Trizol^TM^ reagent (ThermoFisher Scientific, Waltham, MA, USA) using a mechanical homogenizer for RNA isolation. RNA was extracted according to the manufacturer's instructions. For the cDNA synthesis, 500 ng of total RNA was reverse transcribed using PrimeScriptTM RT Master Mix (Perfect Real Time) (RR036A, TaKaRa, Kusatsu, Japan) according to the manufacturer's instructions. Two-step real-time PCR was conducted using Kapa SyBr^®^ Fast Universal kit (KK4618, Merck, Rahway, NJ, USA) on a 7500 Fast Real-Time PCR Instrument (Applied Biosystems^®^, 4351106, Waltham, MA, USA) with 96-well Block Module as follows: start step at 95 °C for 3 min and then 40 cycles of 95 °C for 10 s, and 60 °C for 30 s. All samples were used in technical duplicates, while b-actin and PPIB were used as internal control genes. Primer sequences used are shown in Supplemental table 1. Data analysis was performed using mRNA levels expressed as relative quantification (RQ) values, which were calculated as RQ = 2(–DDCt), where DCt is [Ct (gene of interest)—Ct (housekeeping gene)].

### 2.5 Serum isolation and measurement of biomarkers

For serum collection, mice were anesthetized under continuous flow of isoflurane and blood was collected via cardiac puncture using a 30G needle. Mice were subsequently sacrificed and tissues were collected. Blood was allowed to clot for 10 min and then it was centrifuged at 13,000 rpm for 10 min at 4°C. Serum was collected and stored in −80°C before further analysis. Cytokine concentration of mouse IL-6 (431301, ELISA Max™ Delux Set BioLegent, San Diego, CA, USA) was measured according to the manufacturer's instructions. Samples were diluted 1:2 with 1x assay diluent (provided by the kit) to minimize serum inhibition as recommended by the manufacturer. Dilution was chosen after a trial with serial dilutions was performed. All samples and standards were measured in duplicates, and the absorbance was measured using a microplate reader (51119100, Thermo Scientific™ Multiskan). Absorbance at 570 nm was subtracted from the absorbance at 450 nm. Biochemical parameters (Cholesterol, LDL, HDL, AST, LDH) were measured in an automated biochemical analyser (Beckman AU5800).

### 2.6 Intestinal microbiome sequencing and bioinformatic analysis

Following animal sacrifice, fecal samples were collected from the large intestine and caecum pooled among the two sites of collection and used for microbiome analysis. DNA was extracted with the Nucleospin DNA stool kit (74047250, Macherey-Nagel, Düren, Germany) according to the manufacturer's instructions. For bacterial community assessment, the 16S rRNA gene was employed through sequencing of the V3-V4 hypervariable regions. Library construction followed ‘Illumina's 16S Metagenomic Sequencing Library Preparation' (15044223B) manual. The V3–V4 regions were amplified with the primers described in Klindworth et al. (18), modified to include an Illumina adapter sequence at their 5′ end. The sequences of the forward and reverse primers used for V3-V4 hypervariable regions' amplification were 5′-TCG TCG GCA GCG TCA GAT GTG TAT AAG AGA CAG CCT ACG GGN GGC WGC AG-3′ and 5′-GTC TCG TGG GCT CGG AGA TGT GTA TAA GAG ACA GGA CTA CHV GGG TAT CTA ATC C-3′, respectively. Libraries were evaluated in terms of size and quality on the 5,200 Fragment Analyzer system (Agilent Technologies Inc., Santa Clara, CA, USA) using the DNF-474-0500 kit (Agilent Technologies, Santa Clara, CA, USA). Sequencing was performed on a MiSeq platform (Illumina Inc., San Diego, CA, USA) with the MiSeq^®^ reagent kit v3.

The bioinformatic analysis was conducted with QIIME2 (19). Raw reads were im-ported into the program and removal of adapter sequences was performed with the cutadapt plug-in (20). The resulting paired-end sequences were joined with the vsearch plug-in (21) and those with quality score below 28 were discarded. After-wards, the high-quality sequences were dereplicated and clustered into Operational Taxonomic Units (OTUs). Clustering was performed at 99% sequence identity, employing the open-reference method and the SILVA 132 reference database (22). Chimeric sequences were removed from the data. OTU taxonomic assignment was performed through their alignment against the SILVA 132 database at 99% nucleotide homology, with the BLAST plug-in (23). Archaeal, chloroplastic, or mitochondrial OTUs as well as OTUs lacking taxonomic assignment were filtered from the data. The final OTU table was imported in R version 3.6.0 (24) for further analysis and visualization. Species richness and diversity were assessed in each group using the “estimate richness” function from the phyloseq (25) package. To test for statistically significant differences between groups, pairwise Wilcoxon rank sum tests were performed and the *p*-values were adjusted using the Benjamini & Hochberg method.

Prior to beta diversity analysis, the OTU table was filtered to discard all OTUs with relative abundance <0.1% in all samples. The Bray-Curtis distance matrix was calculated at the OTU level for the filtered table using the vegan package (26). Beta diversity was assessed through Principal Coordinates Analysis (PCoA) based on the Bray-Curtis dissimilarity matrix. Permutational Multivariate Analysis of Variance (PERMANOVA) was applied to test for the existence of statistically significant differences between the groups. PERMANOVA was performed on the Bray-Curtis dissimilarity matrix using the “adonis2” function of the vegan package with 999 permutations. Pairwise comparisons were also performed using the “pairwise.adonis2” function of the pairwiseAdonis package (27). Results' visualization was performed using the ampvis2 (28) and ggplot2 (29) packages.

### 2.7 Statistical analysis

**Figure 2:**
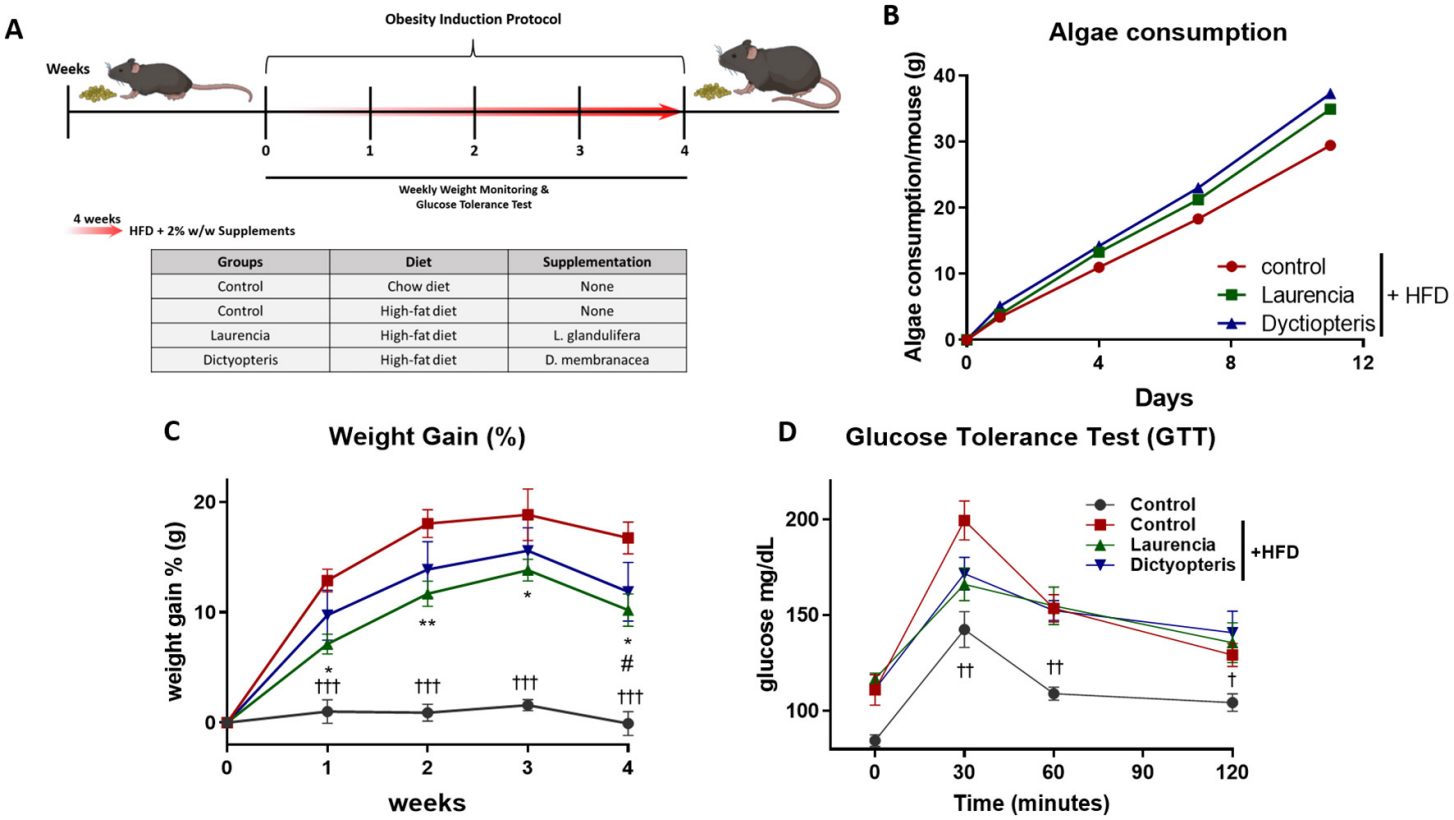
The effect of dried algal biomass consumption on weight gain, glucose intolerance, and inflammation during a 4-week induced obesity protocol. **(A)** Graphical illustration of the experimental design, **(B)** Cumulative food consumption was recorded during a preliminary 12-day cage-based experiment performed prior to the main study to confirm that dietary supplementation with 2% algal biomass did not affect food acceptance. Food intake is presented as cumulative cage consumption per mouse. **(C)** Total body weight change (in grams), **(D)** Glucose tolerance test performed after 3.5 weeks; Two-Way Repeated Measures ANOVA was performed followed by Dunnett's multiple comparisons test, *refers to statistical significance of the HFD control group compared to Laurencia consuming group, ^#^refers to statistical significance of the HFD control group compared to Dictyopteris consuming group of mice and ^‡^refers to chow diet control mice compared to HFD control group. (*n* = 6 mice/group).

Data are presented as mean ± SEM. Graphical design and statistical analysis was performed using Graphpad Prism 7.0. Mann–Whitney t-test as well as one-way ANOVA were performed to determine the statistical significance of differences observed between the groups consuming each supplement and the control high-fat diet consuming group. Multiple comparisons were performed using Dunnett's multiple comparisons test against the control + HFD group. In Figure 2D (GTT) Two-Way Repeated Measures ANOVA was performed followed by Dunnett's multiple comparisons test. Differences with a *p*-value <0.05 were considered significant (^*^indicates *p* < 0.05, ^**^indicates *p* < 0.01, ^***^ indicates *p* < 0.001).

## 3 Results

Previous studies have shown that brominated diterpenes neorogioltriol (1), neorogioldiol (2), and O11,15-cyclo-14-bromo-14,15-dihydrorogiol-3,11-diol (3) isolated from Laurencia sp. and the disulfides bis (5-methylthio-3-oxo-undecyl) disulfide (4), 5-methylthio-1-(3-oxo-undecyl)disulfanylundecan-3-one (5), and 3-hexyl-4,5-dithiocycloheptanone (6) isolated from *D. membranacea* possess anti-inflammatory and analgesic properties in mice (Figure 1) (10, 11, 30). In order to verify the presence of the bioactive compounds 1–6 in the two investigated algae, small quantities of the dried biomass were extracted with organic solvents and the obtained ex-tracts were analyzed by NMR spectroscopy. The acquired 1H NMR spectra revealed characteristic signals confirming the presence of the brominated diterpenes 1–3 in the extract of *L. glandulifera*, whereas in the extract of *D. membranacea* evident were the signals attributed to the disulfides 4–6 (Figure 1). Furthermore, the presence of the bioactive metabolites was verified by chromatographic analysis of the two extracts using the isolated compounds as standards. Due to difficulties in the chemical synthesis of the compounds and increased extraction costs, total dried mass of the algae was used to identify their potential use as dietary supplements during the early stages of high fat diet-induced obesity in mice. Utilizing algae biomass provides the advantage to assess combined and potentially synergistic effects of multiple bioactive constituents, recapitulating traditional dietary habits having physiological relevance.

### 3.1 Diet supplementation with the biomass of *L. glandulifera* or *D. membranacea* suppresses glucose intolerance in high-fat diet-induced mild obese mice

In order to test the potential of the dried algal biomass of *L. glandulifera* and *D. membranacea* as a dietary supplement against hyperglycemia and glucose intolerance, mice were fed with HFD for 8-weeks, representing a fully established obesity model. The results showed that while at the early stages modulated postprandial hyperglycemic spikes in mice fed with the algae, when obesity was fully established this effect was lost (Supplementary Figure S1). Based on these data, the study was designed to focus at the early stages of obesity and glucose intolerance, where the modulatory effects of the algal biomass are more evident. Mice were treated for 4 weeks with HFD supplemented with 2% w/w of dried algal biomass (Figure 2A). This dosage was selected based on established protocols using marine algae supplementation in high-fat diet mouse models for metabolic and microbiome research, where 1%−5% w/w dry algae in feed is common to achieve bioactivity without toxicity, and reflects the percentage of dietary supplement intake in humans (17). In each diet group, 6 mice of both sexes were randomly allocated. In order to verify that mice tolerated the dietary supplements containing the algae and that there is no effect on feed consumption, we conducted a preliminary experiment measuring the amount of supplemented feed the mice were consuming per cage and no differences were observed compared to mice receiving chow diet (Figure 2B). To test the effect of dietary supplementation with *Laurencia* or *Dictyopteris*, mice fed with chow diet (hereafter referred to as control) and a second group was fed with HFD-feed and no other supplement, which was used as control to the groups receiving the supplement (hereafter referred to as control + HFD). Animal weight was monitored weekly and glucose tolerance test was performed at 3.5 weeks, 3 days prior to the termination of the experiment (Figures 2C and Supplementary Figure S2).

**Figure 3:**
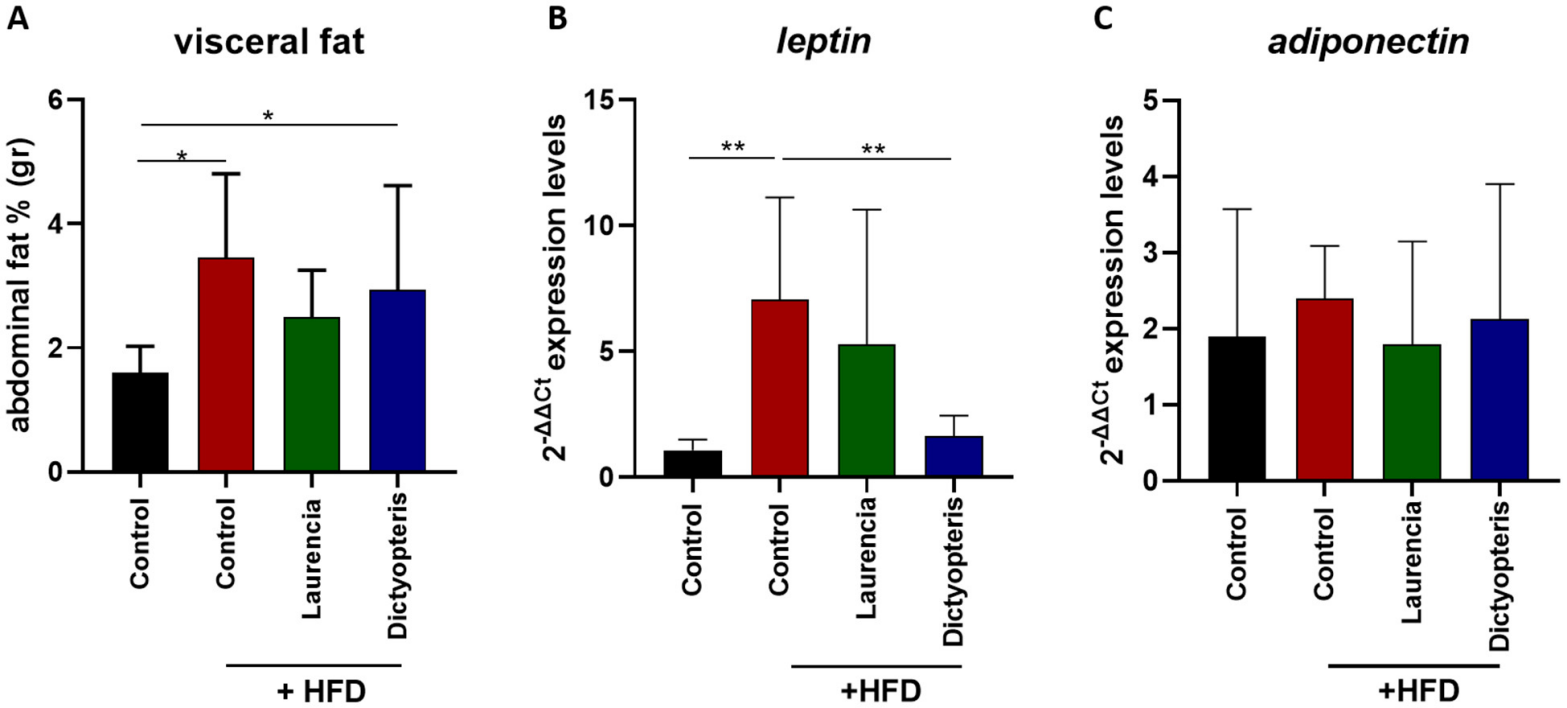
**(A)** Abdominal fat in grams normalized to total body weight, **(B)** Leptin mRNA expression levels, **(C)** Adiponectin mRNA expression levels. Statistical significance has been assessed using one-way ANOVA with Dunnett's multiple comparison test. *p < 0.05, **p < 0.01, (*n* = 6 mice/group).

Interestingly, two-way repeated measures ANOVA revealed a significant treatment × time interaction (*p* = 0.0316), indicating differences in glucose response profiles over time among the experimental groups. Although *post hoc* comparisons between the algae-supplemented groups and the HFD group were not statistically significant after correction for multiple comparisons, both algae exhibited a trend toward attenuation of the early postprandial glucose spikes (Figure 2D). Indeed, 30 min following dextrose administration, blood glucose levels were mildly reduced in these groups compared to the HFD-consuming group indicating that these algae may moderately prevent acute hyperglycemic spikes. To support further this hypothesis, the area under the curve (AUC) of the glucose tolerance test was calculated, showing that total glucose exposure was modestly reduced but not significantly during the 120-minute duration of the experiment (Supplementary Figure S3). Notably, only the group consuming *L. glandulifera* exhibited reduced weight gain (Figure 2C) during the 4-week protocol, consistent with the fact that the same group did not exhibit significant accumulation of visceral fat compared to chow diet control (Figure 3).

To assess the effect of dietary supplementation with these algae on the adipose tissue inflammation, visceral adipose tissue was collected, which was further used to determine the levels of metabolic and inflammatory mediators. Mice fed with a HFD demonstrated increased body weight and visceral adipose tissue compared to the control group (Figures 2C, 3A). Even though supplementation with *Laurencia* significantly reduced weight gain in HFD-fed mice (Figure 2C), neither *Laurencia* nor *Dictyopteris* supplementation in the diet significantly reduced visceral adipose tissue accumulation, compared to HFD control mice (Figure 3A).

As expected, experimental mice consuming HFD exhibited significantly elevated levels of leptin as compared to the control group (Figure 3B), whereas in mice fed with HFD supplemented with *Laurencia* did not exhibit significant changes in leptin levels. In contrast, *Dictyopteris* supplementation significantly reduced leptin expression compared to the HFD control group, indicating a potential beneficial role in energy homeostasis and metabolism (Figure 3B). No significant differences in adiponectin expression were observed between any of the groups (Figure 3C), which was due to the short duration of the obesity, being at the early stages of obesity, where metabolic disturbance occurs, adiponectin is modestly increased as a feedback mechanism to suppress metabolic inflammation (31). In addition, the levels of total cholesterol, LDL and HDL were measured in the serum of mice, showing that the 4-week HFD protocol successfully elevated total cholesterol compared to chow diet controls, but it did not in either group consuming algae (Supplementary Figure S4). Both HDL and LDL were marginally elevated all groups consuming high fat diet (Supplementary Figure S4). To determine the potential effect of obesity and dietary supplementation with the algae in liver integrity, serum levels of the liver enzyme Aspartate aminostrasnferase (AST) and the tissue injury marker Lactate Dehydrogenase (LDH) were measured. No difference was observed indicating that the model did not affect liver function and that both algae did not exhibit any toxicity (Supplementary Figure S4).

**Figure 4:**
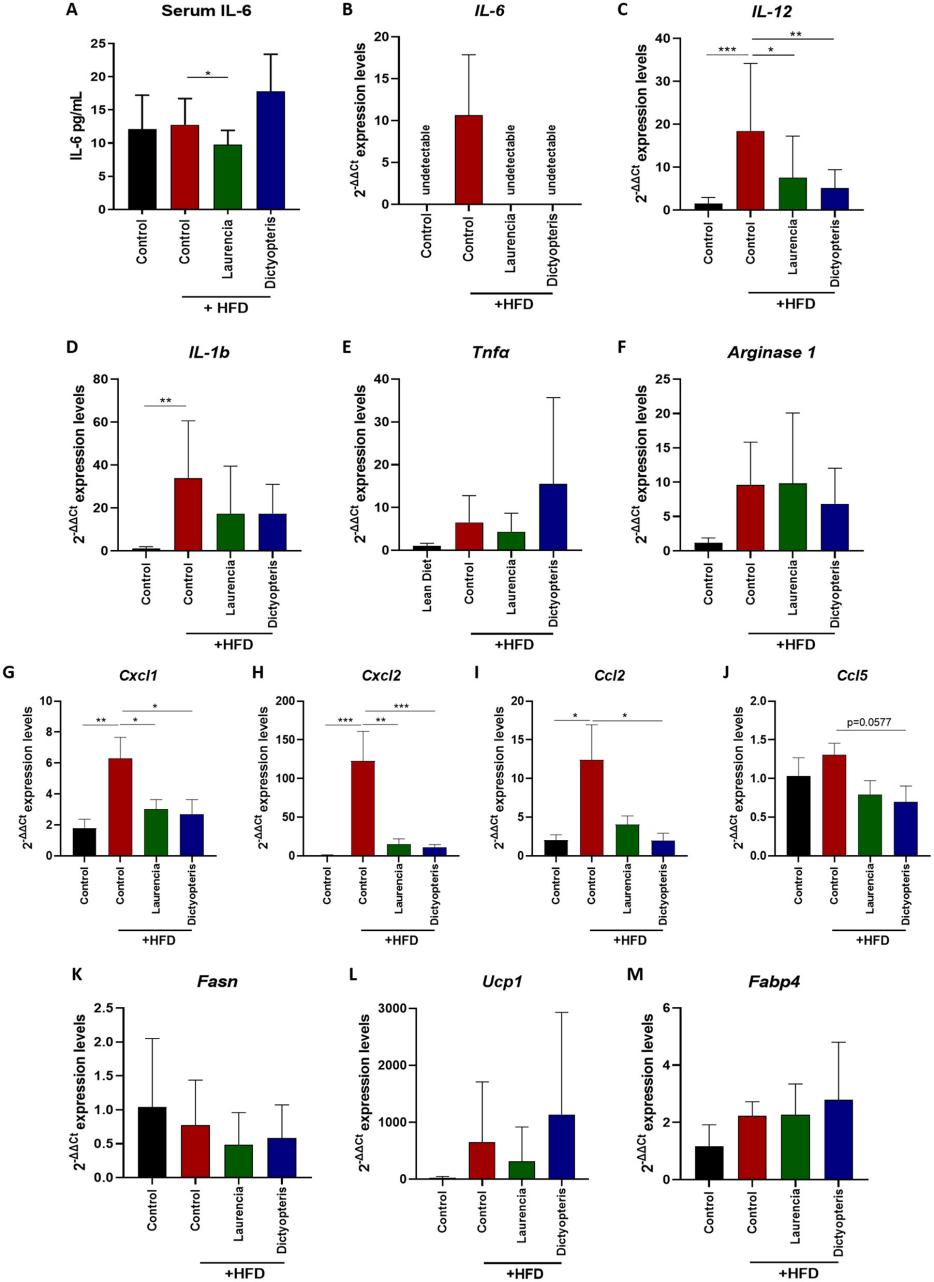
Expression profiling of major inflammatory mediators in the abdominal fat tissue and serum. **(A)** serum IL-6, **(B–J)** mRNA expression levels of inflammatory mediators in abdominal fat tissue. Expression profiling of fatty acid metabolism genes in abdominal fat tissue. mRNA expression levels of **(K)** FasN, **(L)** Ucp1, and **(M)** FABP4. Statistical significance has been assessed using one-way ANOVA with Dunnett's multiple comparison test. *p < 0.05, **p < 0.01, ***p < 0.001 (*n* = 6 mice/group).

It has been well established that obesity and type 2 diabetes result in a chronic low-grade inflammation. Adipose tissue macrophages undergo transcriptional changes inducing a pro-inflammatory M1-like phenotype, producing pro-inflammatory cytokines and chemokines such as TNFα, IL-6, and MCP-1, inducing systemic insulin resistance (32). To investigate whether the underlying mechanism of action involved in *Laurencia* or *Dictyopteris* in increasing insulin sensitivity is due to altered pro-inflammatory cytokine expression in the adipose tissue, mRNA levels of key inflammatory cytokines were measured in the adipose tissue (Figure 4). Although no difference was observed in serum IL-6 levels between control and HFD groups, the group that consumed the Laurencia-supplemented diet mildly decreased IL-6 levels in the serum compared to the HFD control group (Figure 4A), although it should be treated with caution as it falls low in the detection limit of the assay. Subsequently, we extracted RNA from the visceral fat tissue and measured inflammatory cytokine expression using qPCR. Interestingly, expression of IL-6 was undetectable in the adipose tissue of chow diet-fed mice, as well as in both experimental groups consuming the algal biomass-supplemented diets (Figure 4B) within the defined Ct threshold of the 40 cycles of qPCR. This is in accordance to the finding that the *Laurencia*-consuming mice exhibited reduced elevation of IL-6 in both serum and visceral fat tissue, whereas the *Dictyopteris*-consuming mice maintained undetectable IL-6 expression only in the visceral fat tissue. These results were further supported as both groups consuming the algal biomass-supplemented diets showed significantly decreased expression of IL-12 (Figure 4C), a key pro-inflammatory cytokine implicated in metabolic dysregulation during obesity (33). Although IL-1b and TNFα are also key pro-inflammatory cytokines produced and secreted by the adipose tissue during obesity, no statistically significant differences were observed in the HFD-consuming groups (Figures 4D, E), suggesting that they were not affected in our model.

Aiming to further investigate the potential role of dietary supplementation with the algal biomass in the expression of anti-inflammatory mediators, we measured the expression levels of Arginase 1 but no statistically significant differences were observed (Figure 4F). No statistically significant differences were observed between the chow diet group and the HFD group concerning TNFα (Figure 4E), whereas protein levels of TNFα were found to be below detection limit in serum using Elisa, supporting the fact that our model represents early stages of obesity development rather than established obesity. To further evaluate changes in chemokines that are central in recruiting inflammatory cells, we measured the expression of Chemokine C-X-C motif ligand (cxcl1, cxcl2, C-C motif chemokine ligand (ccl) 2 and ccl5 in the visceral fat of mice supplemented with either alga. The results showed reduction in the expression of cxcl1 and cxcl2 following supplementation of *Laurencia* or *Dictyopteris* and suppression of ccl2 following supplementation with *Dictyopteris* (Figures 4F–J), suggesting a potential suppression of inflammatory cell recruitment in the visceral fat tissue, even though further histological evidence is needed to assess cellular infiltration. Overall, consumption of diets supplemented with *Laurencia* or *Dictyopteris* decreased the expression of pro-inflammatory cytokines IL-6 and IL-12, and chemokines cxcl1, cxcl2 and ccl2, indicating a potential anti-inflammatory role of both algae in the adipose tissue. Further aiming to investigate the possible role of the supplementation with the algal biomass in reshaping adipose tissue metabolism, we measured the expression levels of FasN, Ucp1, and FABP4 using qPCR (Figures 4G–I). No statistically significant differences in the expression levels were observed between the different groups, also reflecting that the model represents early stages of obesity with modest metabolic dysregulation.

### 3.2 Diet supplementation with the biomass of *L. glandulifera* or *D. membranacea* modulates intestinal microbiome consistency

Tissues exposed to the environment such as the skin, mouth, gut, and vagina, are colonized by microorganisms, consisting the local tissue microbiome. The intestinal microbiome is altered in obesity and has been highlighted as a critical contributor to the development of obesity, diabetes, and metabolic syndrome (34, 35).

**Figure 5:**
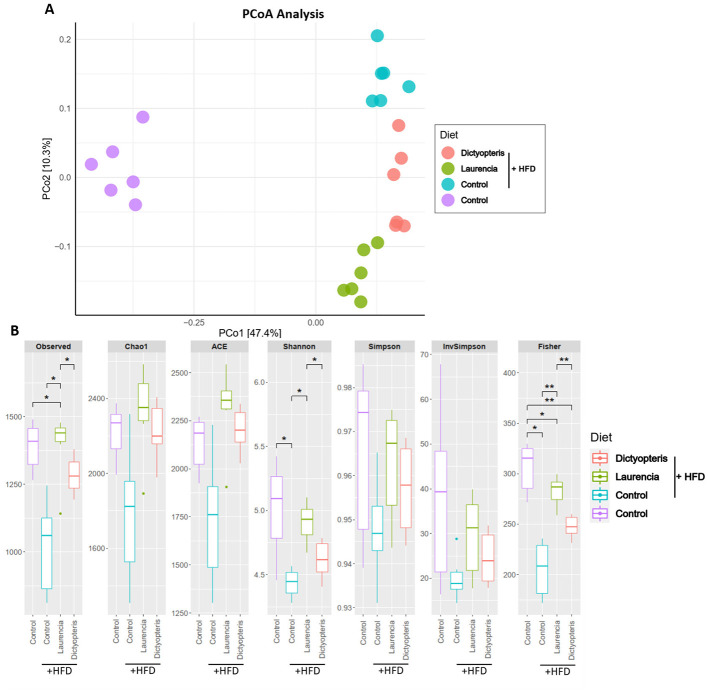
The effect of the algal biomass-supplemented diet consumption on the gut bacterial communities. **(A)** PCoA analysis and **(B)** α-diversity analysis. *p < 0.05, **p < 0.01 (*n* = 6 mice/group).

Accordingly, we investigated whether diet supplementation with the algal biomass would affect intestinal microbiome composition and whether this could result in reduced inflammation during obesity. To test our hypothesis, we fed the mice for 4 weeks with HFD supplemented with 2% (w/w) of the dried biomass of *L. glandulifera* or *D. membranacea* and then collected fecal samples from the cecum and the large intestine. DNA was extracted and a microbiome analysis was performed through 16S rRNA sequencing. A Principal Coordinates Analysis (PCoA) was conducted based on the identified OTUs to assess genetic clustering of the different diet groups (Figure 5A). Four distinct groups were observed, one including the control mice, one the mice fed with HFD alone and one for each group receiving algal dietary supplementation (Figure 5A). As expected, the PCoA showed significant differences using Permanova between the chow diet (control) and HFD groups (Supplementary Figure S5). Interestingly, the groups supplemented with the algal biomass clustered separately and at a distance from the HFD control group, pointing to a different bacterial community composition. The PCoA clustering was also verified by the α-diversity indices.

As expected, independently of the index studied (Chao1, ACE, Shannon, Simpson, Inverse Simpson, and Fisher) the chow diet-fed group (control) exhibited higher diversity in terms of species richness (Figure 5B) as limited microbiome diversity is associated with the obese phenotype (36). Importantly, the *Laurencia* diet-fed mice exhibited no difference in the microbiome diversity compared to the chow diet-fed mice, further supporting its role in restoring the gut microbiome composition and reducing inflammation. Moreover, supplementation with *Laurencia* slightly elevated the presence of rare microbial taxa. Concerning the diet supplemented with *Dictyopteris*, it also reversed the limited microbiome diversity observed during obesity, but to a lesser extent.

**Figure 6:**
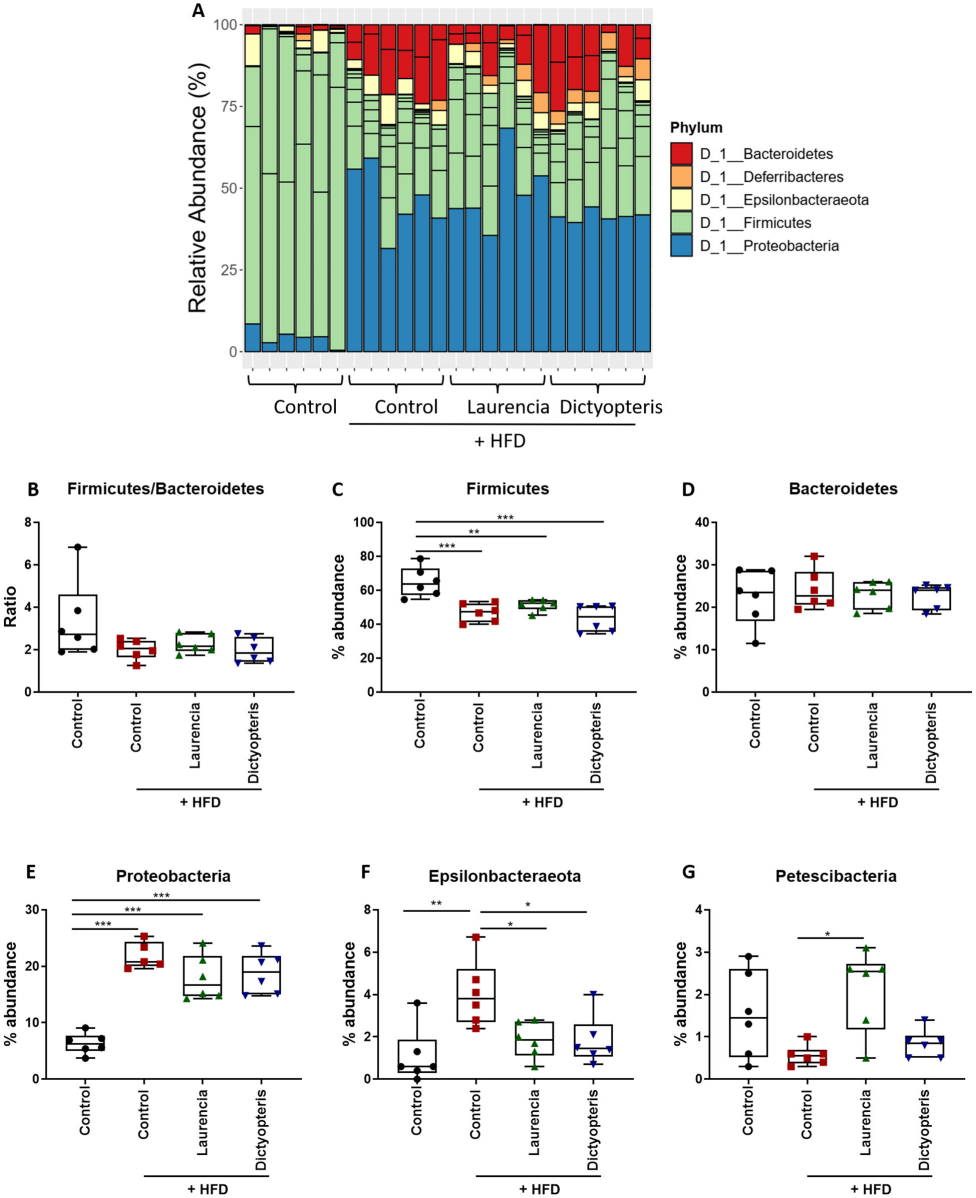
The effect of administration of the algal biomass-supplemented diets on the gut microbiome's top phyla abundance levels. **(A)** Barplot of normalized phyla abundances (%) based on the identified OTUs, **(B–G)** Boxplots presenting the abundance of different phyla following diet supplementation. Statistical significance has been assessed using one-way ANOVA with Dunnett's multiple comparison test. *p < 0.05, **p < 0.01, ***p < 0.001 (*n* = 6 mice/group).

Investigation of the bacterial communities' composition at the phylum level revealed the most abundant microbial phyla (Figure 6A) and further analyses showed that for the ratio Firmicutes/Bacteroidetes, that is widely accepted as a marker of dysbiosis [specifically, increased ratio is observed upon the induction of obesity (37)], no statistically significant difference was observed (Figure 6B), indicating that dysbiosis was not fully established during the 4-week experimental protocol. Among the most abundant phyla, Firmicutes (Figure 6C), Bacteroidetes (Figure 6D), and Proteobacteria, only Proteobacteria were found to be significantly reduced in the algae-consuming groups (Figure 6E), indicating restricted microbiota changes, as Proteobacteria expansion during obesity compromises the ability to maintain a balanced gut microbial community (38). Likewise, comparing less abundant microbial phyla, the algae-consuming groups exhibited reduced colonization of the Epsilonbacteraeota phylum (synonym Campylobacteriota and Epsilonproteobacteria) as compared to the HFD control group and even same levels to the control chow diet-fed mice (Figure 6F). In the same context, Laurencia-consuming mice showed significantly elevated -compared to the HFD control- colonization by the phylum Petescibacteria (Figure 6G).

**Figure 7:**
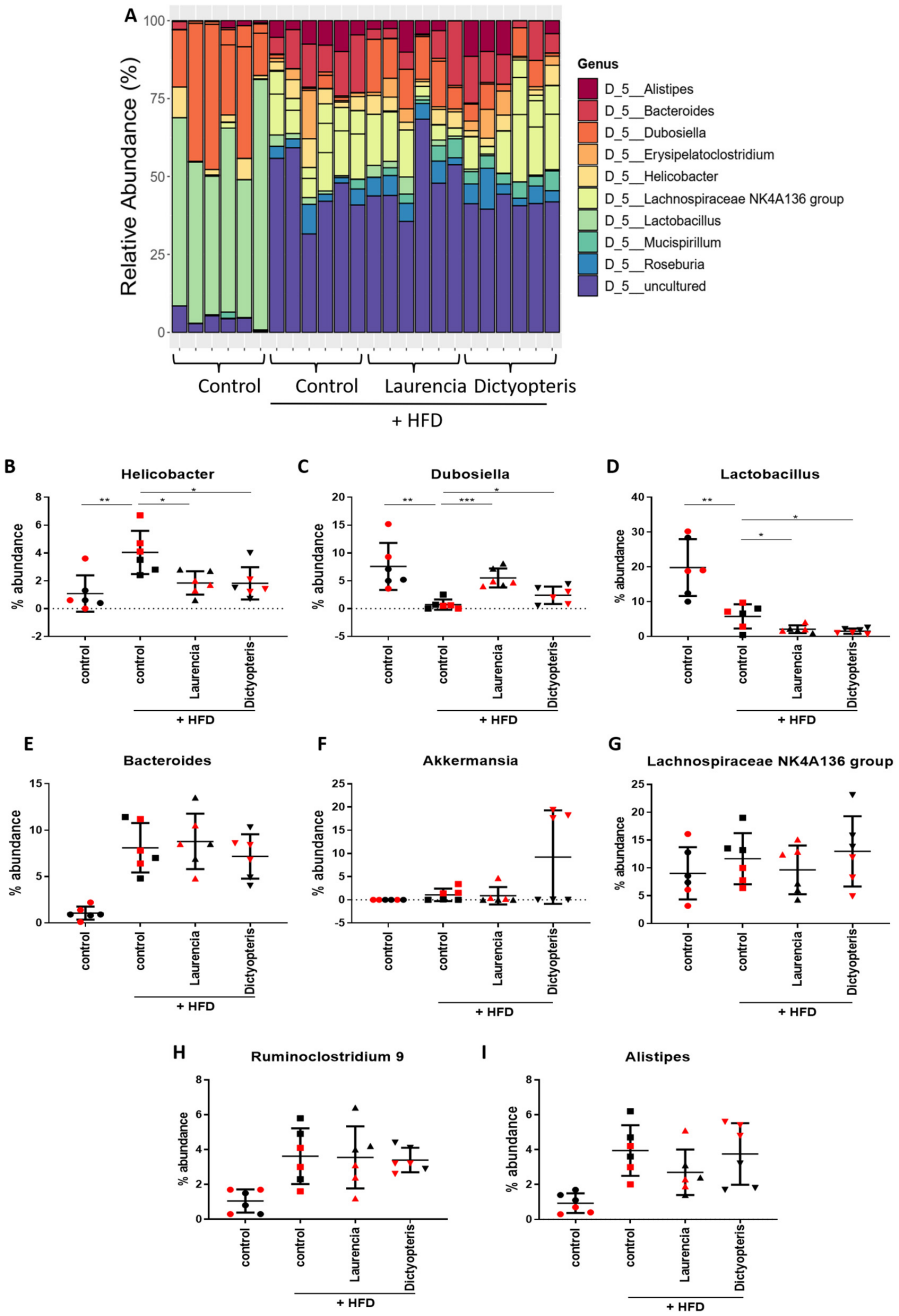
The effect of administration of the algal biomass-supplemented diets on the gut microbiome's top genera abundance levels. **(A)** Barplot of normalized genera abundances (%) based on the identified OTUs, **(B–I)** Boxplots presenting the abundance of different genera following diet supplementation. Red dots represent female mice and black dots male mice. Statistical significance has been assessed using one-way ANOVA with Dunnett's multiple comparison test. *p < 0.05, **p < 0.01, ***p < 0.001 (*n* = 6 mice/group).

Further investigation of the microbial communities' composition at the genus level showed that HFD-consuming groups, both control and algae-consuming, were characterized by a larger fraction of uncultured bacteria compared to the chow diet-fed mice (Figure 7A). Specifically, algae supplementation attenuated colonization of the Helicobacter genus (Figure 7B), which has been shown to correlate with the obese phenotype, as well as to induce gastric inflammation through its most common species, Helicobacter pylori (39, 40). In addition, diet supplementation with *Laurencia*, as well as with Dictyopteris, increased the abundance of the genus Dubosiella (Figure 7C) compared to the HFD diet. Nevertheless, administration of both algae reduced the Lactobacillus genus colonization (Figure 7D), even though it is a well-established member of the beneficial microbial community of the gut. Furthermore, genera Bacteroides, Akkermansia, Lachnospiraceae NK4A136 group, Ruminoclostridium 9 and Alistipes (Figures 7E–I) did not exhibit any statistical differences among the groups receiving the algal supplements.

In this study, we included mice of both sexes to evaluate sex-independent effects in glucose intolerance and the gut microbiome. However, after performing an exploratory analysis of microbial sequencing data in a sex-dependent perspective, we came upon some very interesting observations. Specifically, algae supplementation modulated in a sex-dependent manner the abundance of specific beneficial genera. Laurencia supplementation induced a positive trend the abundance of the Lachnospiraceae NK4A136 group in female mice, and also had an opposite effect in male mice (Supplementary Figure S1). Similarly, an opposite activity in male mice, was observed for the Alistipes genus found in healthy individuals, when mice consumed Dictyopteris, whereas its consumption induced a positive trend in Akkermasia colonization only in female mice (Supplementary Figure S6), highlighting the interplay of sex with the gut microbiome and the response to certain diets. Being an interesting observation and given the limited number of animals per sex, further investigation is necessary to highlight the extent of the sex-specific changes in the gut microbiome conferred by dietary supplements.

## 4 Discussion

The present study investigated the potential use of marine macroalgae *Laurencia glandulifera* and *Dictyopteris membranacea* as dietary supplements, using a model of early-stage high-fat diet (HFD)-induced obesity and glucose intolerance. The model of a short-term HFD-induced obesity was used to determine the effect of the supplementation during the early stages of metabolic dysregulation and at the point when glucose intolerance is being developed, to evaluate whether diet supplementation with algae biomass can be preventive to the development of glucose intolerance. In addition, this model provides the advantage to monitor the impact on the disease when glucose intolerance has been established without having additional pathologies established, which complicate the mechanism. Key findings of the study include the modest improvement of glucose intolerance, reduction of visceral adipose tissue cytokine and chemokine expression and modulation of intestinal microbial composition.

Previous analyses of samples from these algae, have shown that they are a rich source of protein and carbohydrates (40% and 30% of dry mass, respectively) and contain several bioactive molecules including brominated diterpenes and disulfides. Their concentration ranged between 0.9%−1.2% w/w for the brominated diterpenes and 0.08%−0.11% w/w for the disulfides in the dry biomass of *L. glandulifera* and *D. membranacea*, respectively, variations that depend on the season and the year of collection. In addition to these metabolites macroalgae are well-recognized sources of other nutrients that present bioactivity. Among those, bioactive polysaccharides have been shown to possess metabolic and anti-inflammatory effects (41, 42). Red algae contain sulfated galactans, whereas brown algae are rich in alginate and fucoidan, known to possess anti-inflammatory and anti-diabetic properties through multiple mechanisms including NF-kB signaling attenuation, macrophage infiltration inhibition and reduction of insulin resistance (42). Given that obesity is a state of chronic low-grade inflammation and the fact that whole algal biomass was administered, it is plausible that beneficial effects are likely mediated by synergistic actions among multiple classes of bioactive compounds, including terpenoids, disulfides, and polysaccharides.

White adipose tissue and particularly visceral adipose tissue, is an interactive endocrine organ, consisting mainly of adipocytes and the stromal vascular fraction that includes adipose tissue macrophages (ATMs), endothelial cells, and other immune cells such as T cells and neutrophils (30). Adipose tissue contributes to multiple homeostatic processes, Including metabolism, energy storage and release, regulation of glucose and cholesterol, production of molecules that control hunger and satiety, and also contributes to immunity by releasing adipokines, fatty acids, and inflammatory cytokines (43). Indeed, obesity-induced type 2 diabetes is associated with the synthesis of pro-inflammatory cytokines, leading to chronic low-grade inflammation that can result in macrophage infiltration in the adipose tissue, further promoting insulin resistance and glucose intolerance (3). Adipocytes, as well as adipose tissue macrophages, are a major source of cytokines in the adipose tissue during obesity. In lean individuals, M2 polarized adipose tissue macrophages acquire an M2-like phenotype secreting anti-inflammatory mediators such as IL-10 and IL-1 receptor antagonists, to induce insulin sensitivity and maintain homeostasis (44). In obesity, adipose tissue macrophages undergo transcriptional changes inducing a pro-inflammatory M1-like phenotype, producing pro-inflammatory cytokines and chemokines such as TNFα, IL-6, and MCP-1, inducing systemic insulin resistance (32).

Diet supplementation with *L. glandulifera* attenuated early postprandial glucose spikes 30 minutes after dextrose administration, as well as, reduced weight gain independent of feed consumption. In agreement, *L. glandulifera* consuming mice accumulated less visceral fat compared to chow diet control, although this was independent of adipokine expression. We propose that this could be partially explained by the fact that it has been shown that *Laurencia* secondary metabolites modulate adipocyte differentiation and lipid accumulation in mice (patent WO_2025104209_A1). Importantly, *Laurencia* moderately prevented the increase of pro-inflammatory cytokine expression of *il-6* and *il-12* as well as chemokines *cxcl1* and *cxcl2* indicating a potential anti-inflammatory role of the alga in the visceral fat tissue. Moreover, the supplementation with *D. membranacea* resulted in mild reduction of early postprandial glucose spikes, but this was not due to changes in visceral fat accumulation although leptin expression levels were found to be reduced in the visceral fat tissue. Leptin is essential to regulate food intake and body weight by promoting lipid oxidation, and increased leptin levels are associated with obesity due to the development of central leptin resistance (45). It also acts as a pro-inflammatory molecule and chronically elevated leptin levels decrease responsiveness of pancreatic β-cells, leading to hyperinsulinemia and insulin resistance (45). Fatty Acid Synthase (FasN), uncoupling protein 1 (Ucp1), and fatty acid binding protein 4 (FABP4) are genes associated with the accumulation of fatty acids and their metabolism. During obesity, FasN expression is known to increase as a result of excess energy intake, contributing to storage of excess fatty acids, whereas Ucp1 is a mitochondrial uncoupling protein that is stimulated by long chain fatty acids in brown adipose tissue to generate heat in mammalian non-shivering thermogenesis (46). FABP4 is an intracellular lipid chaperone involved in lipid transport, that also acts as an adipokine in the liver, promoting insulin resistance during obesity (47). Both algae failed to induce any statistical differences in the expression profile of fatty acid metabolism genes UCP1, FasN and FABP4 further strengthening the fact that limited metabolic dysregulation occurs in the mild obesity protocol. Importantly, *D. membranacea* significantly reduced the expression of chemokines *cxcl1, cxcl2, ccl2* and marginally *ccl5* suggesting a potential attenuation of inflammatory cell recruitment in adipose tissue, however, direct assessment of immune cell infiltration in the visceral fat was not evaluated. Therefore, mechanistic interpretations should remain cautious and be considered as the seed of further research exploring the molecular mechanism of action.

The gut is primarily colonized by symbiotic bacteria. Certain commensal bacteria are known to exert beneficial effects on the host, such as limiting colonization of potentially pathogenic microorganisms, providing nutrients, metabolizing undigested nutrients, and even shaping the development and function of the immune system, frequently mediated through the production of short chain fatty acids (SCFAs) (48, 49). The gastrointestinal microbial composition and host immune tolerance are subjected to a continuous interplay. However, microbial imbalance can be initiated by various factors including antibiotic consumption, the diet, physical and psychological stress, resulting in gut enrichment with opportunistic microorganisms impairing the host's wellness and homeostasis (50). In the present study, intestinal microbiome analysis indicated that dietary supplementation was associated with changes in microbial community structure in presence of HFD. However, the absence of significant differences in the Firmicutes/Bacteroidetes ratio, together with the short duration of the experimental model, suggests that a fully established dysbiotic state was not achieved. Consequently, the findings are more appropriately interpreted as modulation of the gut microbiota at an early-stage obesity and glucose intolerance context. Changes in specific microbial taxa, including *Helicobacter* and *Dubosiella*, were observed highlighting the potential beneficial role of diet supplementation with the algae. Dubosiella exhibits significant anti-inflammatory properties, managing oxidative stress, and positively regulating vascular endothelial function during inflammation (51), and it is known for its prebiotic properties, being a member of SCFA producers (52). The observed reduction in *Lactobacillus* abundance following algal supplementation may initially appear adverse, given its widely recognized beneficial. However, similar decreases *in Lactobacillus* abundance have been reported in high-fat diet models (53), suggesting that these changes may indicate that intestinal microbial colonization is a constant dynamic process driving the reduction of certain species over the dominance of more adaptive ones. Sex differences in intestinal microbiome composition were observed in the present study, in accordance with previous studies (54), however, these differences should be interpreted with caution due to limited sample size. Nevertheless, a trend toward distinct sex-dependent colonization profiles were observed concerning Akkermansia, Alistipes and Lachnospiraceae NK4A136 genera being important candidates for further research, as they may provide valuable insights into sex-specific host microbiome.

Several limitations should be mentioned concerning the present study, which can be the seed for further research. Despite the fact that, short-term high-fat diet feeding models, including 4-week protocols, are widely used to investigate the early stages of obesity-associated hyperglycemia, glucose intolerance and early metabolic dysfunction in mice (14, 55, 56) the short duration of the HFD protocol reflects early metabolic alterations rather that a fully obese phenotype, thus this study does not recapitulate metabolic changes that occur in fully established type-2 diabetes or obesity. In addition, the rather small sample size, although it fully reflects the 3Rs in laboratory animal handling may limit statistical power and contribute to variability in some results. Both male and female mice have been shown to develop glucose dysregulation, and metabolic inflammation following high-fat diet feeding, even though sex-dependent differences in inflammatory responses have been reported (57, 58). The sex-dependent action is minimal in young adult mice (8 week old mice) while it is more profound in old (32 week old) or juvenile (5 week old) mice (59). In our study we used 8-week old mice to allow drawing conclusions that are sex-independent. Although both sexes of mice were included in the study, the small number of mice limits the ability to draw definitive conclusions regarding sex-dependent effects, but our observations may be a useful basis for further studies. It should be noted that although mice in the present study exhibit a glucose intolerant phenotype, key metabolic parameters, including circulating insulin levels and direct assessments of insulin sensitivity, were not evaluated due to limited volume of serum available. Additionally, although hepatic lipid accumulation may influence glucose metabolism, due to the short duration of the model, significant hepatic alterations are not expected at this stage, as supported by the liver homeostasis parameters measured (Supplementary Figure S4). Inflammatory markers were found to be differentially expressed but further elucidation measuring protein levels could be of great value. An additional limitation in the present study is that dietary supplementation was performed using whole dried algal biomass, and although it serves the purpose of studying the effect of whole algae as dietary supplements, it includes proteins, polysaccharides, dietary fiber, minerals and other bioactive constituents that may have contributed to the observed metabolic and microbiome effects, rather than the identified brominated diterpenes we have previously identified. Furthermore, chemical composition of wild macroalgae may vary according to seasonal changes, geographical location and environmental conditions, highlighting the importance of future standardization of algal biomass production through standardized cultivation methods and batch-to-batch quality control. Finally, intestinal microbiome abundances possess great interest but functional measurements of SCFAs, effectors of the microbiota interplay with the immune and metabolic systems would complement the presented data providing potential mediators of the algae actions.

## 5 Conclusions

This work demonstrates the critical role of marine macroalgae as a potential dietary supplement that can contribute to the reduction of early metabolic dysfunction. Diet supplementation with *L. glandulifera* and *D. membranacea* in a short-term high fat diet model ameliorated early postprandial glucose spikes, compared to control, reduced the expression of proinflammatory cytokines and chemokines, whereas the group that received dietary supplement with *L. glandulifera* gained less weight compared to control mice during the obesity induction protocol. Moreover, the intestinal microbial dysregulation arising from the consumption of a diet high in lipids and the systemic metabolic imbalance, was partly reversed by both algae, partially restoring microbial colonization and microbial abundance. These findings have revealed the potential of the red alga *L. glandulifera* and the brown alga *D. membranacea* as a source of nutrients that can modulate metabolic dysfunction at the early stages of obesity and improve intestinal microbial composition in mice. Importantly, these two algae are traditionally consumed as a delicacy by locals of the coastal regions at Kefalonia and Ithaca islands Greece, respectively, offer a promising strategy to prevent diet-induced glucose intolerance and metabolic inflammation and restore gut microbial homeostasis.

## Data Availability

The datasets presented in this study can be found online in https://zenodo.org/records/14536557.
