# Supplementary material for: Diet supplementation with the macroalgae Laurencia glandulifera and Dictyopteris membranacea limits postprandial hyperglycemia and alters intestinal microbiome in mice
Source: Front Nutr. 2026 Aug 4;13:1923067. doi: 10.3389/fnut.2026.1923067 (PMC13483065; doi:10.3389/fnut.2026.1923067)
Supplement: Supplementary file 1 [file Data_Sheet_1.docx]

Supplementary Material

| **Oligonucleotide** | **Sequence** |
| --- | --- |
| TNFα-Fwd | GCCCACGTCGTAGCAAACCACC |
| TNFα-Rev | CGGGGCAGCCTTGTCCCTTG |
| IL-6-Fwd | CAAAGCCAGAGTCCTTCAGAG |
| IL-6-Rev | CACTCCTTCTGTGACTCCAGC |
| Arginase 1-Fwd | CAGAAGAATGGAAGAGTCAG |
| Arginase 1-Rev | CAGATATGCAGGGAGTCACC |
| FABP4-Fwd | GCTCCTCCTCGA AGGTTTACA A |
| FABP4-Rev | AATCCCCATTTACGCTGATGAT |
| IL-12b-Fwd | GTAACCAGAAAGGTGCGTTCC |
| Il-12b-Rev | GAACACATGCCCACTTGCTG |
| Leptin-Fwd | TCACACACGCAGTCGGTATC |
| Leptin-Rev | GCACATTTTGGGAAGGCAGG |
| Adiponectin-Fwd | AAAGGAGATGCAGGTCTTCTTG |
| Adiponectin-Rev | CTGAGCGATACACATAAGCGG |
| IL-1β-Fwd | CGGACCCCAAAAGATGAAGGGCTG |
| IL-1β- Rev | GCTCTTGTTGATGTGCTGCTGCGAG |
| FasN-Fwd | GACTCGGCTACTGACACGAC |
| FasN-Rev | CGAGTTGAGCTGGGTTAGGG |
| Ucp1-Fwd | TTCAGGGAGAGAAACACCTGC |
| Ucp1-Rev | ATTGTAGGTCCCCGTGTAGC |
| PPIB-Fwd | ACAGCAAGTTCCATCGTGTCAT |
| PPIB-Rev | GAAGAACTGTGAGCCATTGGTG |
| Actin-Fwd | CATTGCTGACAGGATGCAGAAGG |
| Actin-Rev | TGCTGGAAGGTGGACAGTGAGG |
| Cxcl1-Fwd | ACCCGCTCGCTTCTCTGT |
| Cxcl1-Rev | TCTGAACCAAGGGAGCTTCA |
| Cxcl2-Fwd | CCACTGCGCCCCAGACAGAAG |
| Cxcl2-Rev | AACTTTTTGACCGCCCTTGAG |
| Ccl2-Fwd | ATCACCAGCAGCAGGTGTCC |
| Ccl2-Rev | GAGCTTGGTGACAAAAACTACACC |
| Ccl5-Fwd | TGCTCCAATCTTGCAGTCGT |
| Ccl5-Rev | ACACACTTGGCGGTTCCTTC |

**Table S1.** List of oligonucleotides used in real time PCR reactions

**Supplementary Figure 1.** The effect of dried algal biomass consumption on mice weight during an 8-week induced obesity protocol. Glucose tolerance test performed after 4,6 and 8 weeks of feeding; One-way ANOVA has been performed, (n=5 mice/group).

**Supplementary Figure 2.** The effect of dried algal biomass consumption on mice weight during a 4-week induced obesity protocol. (A) Total body weight (in grams). One-way ANOVA has been performed. Data represent mean ± SEM. N=6 animals/group

**Supplementary Figure 3.** Area under the curve (AUC) of glucose tolerance test performed at 3.5 weeks of feeding the mice with the respected algae. One-way ANOVA has been performed. Data represent mean ± SEM. ***p<0.001; N=6 animals/group

**Supplementary Figure 4.** Lipid profiling and hepatic measurements in animal sera after a 4-week induced obesity protocol. One-way ANOVA has been performed. Data represent mean ± SEM. N=6 animals/group

**Supplementary Figure 5.** Permanova comparisons

**Supplementary Figure 6.** Sex-dependent differences in microbial genera gut colonization depicted using boxplots representing the abundance of different genera following diet supplementation. Red dots represent female mice and black dots male mice. One-way ANOVA and Mann–Whitney t-test were performed. * p < 0.05, ** p < 0.01, *** p < 0.001.
